# Visual perception of light organ patterns in deep‐sea shrimps and implications for conspecific recognition

**DOI:** 10.1002/ece3.6643

**Published:** 2020-08-07

**Authors:** Lorian E. Schweikert, Alexander L. Davis, Sönke Johnsen, Heather D. Bracken‐Grissom

**Affiliations:** ^1^ Department of Biological Sciences and Institute of Environment Florida International University North Miami FL USA; ^2^ Biology Department Duke University Durham NC USA

**Keywords:** bioluminescence, crustacean, sighting distance, spatial resolution, vision, visual acuity

## Abstract

Darkness and low biomass make it challenging for animals to find and identify one another in the deep sea. While spatiotemporal variation in bioluminescence is thought to underlie mate recognition for some species, its role in conspecific recognition remains unclear. The deep‐sea shrimp genus, *Sergestes* sensu lato (s.l.), is one group that is characterized by species‐specific variation in light organ arrangement, providing us the opportunity to test whether organ variation permits recognition to the species level. To test this, we analyzed the visual capabilities of three species of *Sergestes* s.l. in order to (a) test for sexual dimorphism in eye‐to‐body size scaling relationships, (b) model the visual ranges (i.e., sighting distances) over which these shrimps can detect intraspecific bioluminescence, and (c) assess the maximum possible spatial resolution of the eyes of these shrimps to estimate their capacity to distinguish the light organs of each species. Our results showed that relative eye size scaled negatively with body length across species and without sexual dimorphism. Though the three species appear capable of detecting one another's bioluminescence over distances ranging from < 1 to ~6 m, their limited spatial resolution suggests they cannot resolve light organ variation for the purpose of conspecific recognition. Our findings point to factors other than conspecific recognition (e.g., neutral drift, phenotypic constraint) that have led to the extensive diversification of light organs in *Sergestes* s.l and impart caution about interpreting ecological significance of visual characters based on the resolution of human vision. This work provides new insight into deep‐sea animal interaction, supporting the idea that—at least for these mesopelagic shrimps—nonvisual signals may be required for conspecific recognition.

## Graphical abstract

This is the first study to examine if mesopelagic shrimps can visually discriminate variation in light organ arrangement for the purpose of conspecific recognition. This provides new knowledge about deep‐sea animal interaction, ultimately pointing to factors other than conspecific recognition (e.g., drift) that have led to the diversification of bioluminescent light organs.

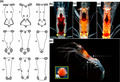

## 1 INTRODUCTION

With few exceptions, sex, sociality, predation, and other behaviors require animals to find and identify one another. In the deep sea (>200 m depth), encountering and recognizing other animals is made difficult by factors including the magnitude of the environment (in both depth and extent), darkness (Warrant & Locket, 2004), and reduced biomass at depth (Herring, 2000). In solving this problem, a variety of bioluminescent signals have evolved among deep‐sea animals (Haddock, Moline, & Case, 2010), yielding morphological, physiological, and behavioral variation in bioluminescence that in some cases is thought to play a role in intraspecific recognition.

Within species, various differences in bioluminescence between sexes can be implicated in mate recognition. For example, these differences can occur temporally, as in some marine ostracods with male courtship displays that are comprised of bioluminescent pulses or flashes (Gerrish & Morin, 2016; Rivers & Morin, 2008). They can also occur spatially, such as sexual dimorphism in the size, shape, and/or arrangement of light organs and their potential role in identifying mates (Herring, 2007). For example, males of the euphausiid crustacean, *Nematobrachion flexipes*, have a prominent abdominal photophore that is absent in females, and among dragonfishes, many species present sexual dimorphism in postorbital light organ size (Badcock & Merrett, 1976; Gibbs, 1969; Herring, 2007; Marshall, 1979). As these sex differences in bioluminescence might underlie mate recognition, species‐specific differences in bioluminescence might underlie conspecific recognition—that is, the capacity of some animals to distinguish hetero‐ from con‐specifics for functions that include the ability to identify either competitors or mates (Okamoto & Grether, 2013). Recent studies of myctophids (lanternfish; Davis, Holcroft, Wiley, Sparks, & Smith, 2014), etmopterids (lanternshark; Claes, Nilsson, Mallefet, & Straube, 2015), and other groups (Ellis & Oakley, 2016) have shown that species‐level variation in bioluminescence is correlated with increased rates of speciation. It is predicted that distinct patterns of bioluminescence act as visual signals that lead to reproductive isolation and increased species richness (Claes et al., 2015; Davis et al., 2014; Ellis & Oakley, 2016). This prediction, however, is dependent on the visual abilities of conspecifics to detect and discriminate differences in bioluminescent pattern, and for many groups with species‐specific differences in bioluminescence, the capacity to perceive the patterns of conspecifics remains unknown.

**FIGURE 1:**
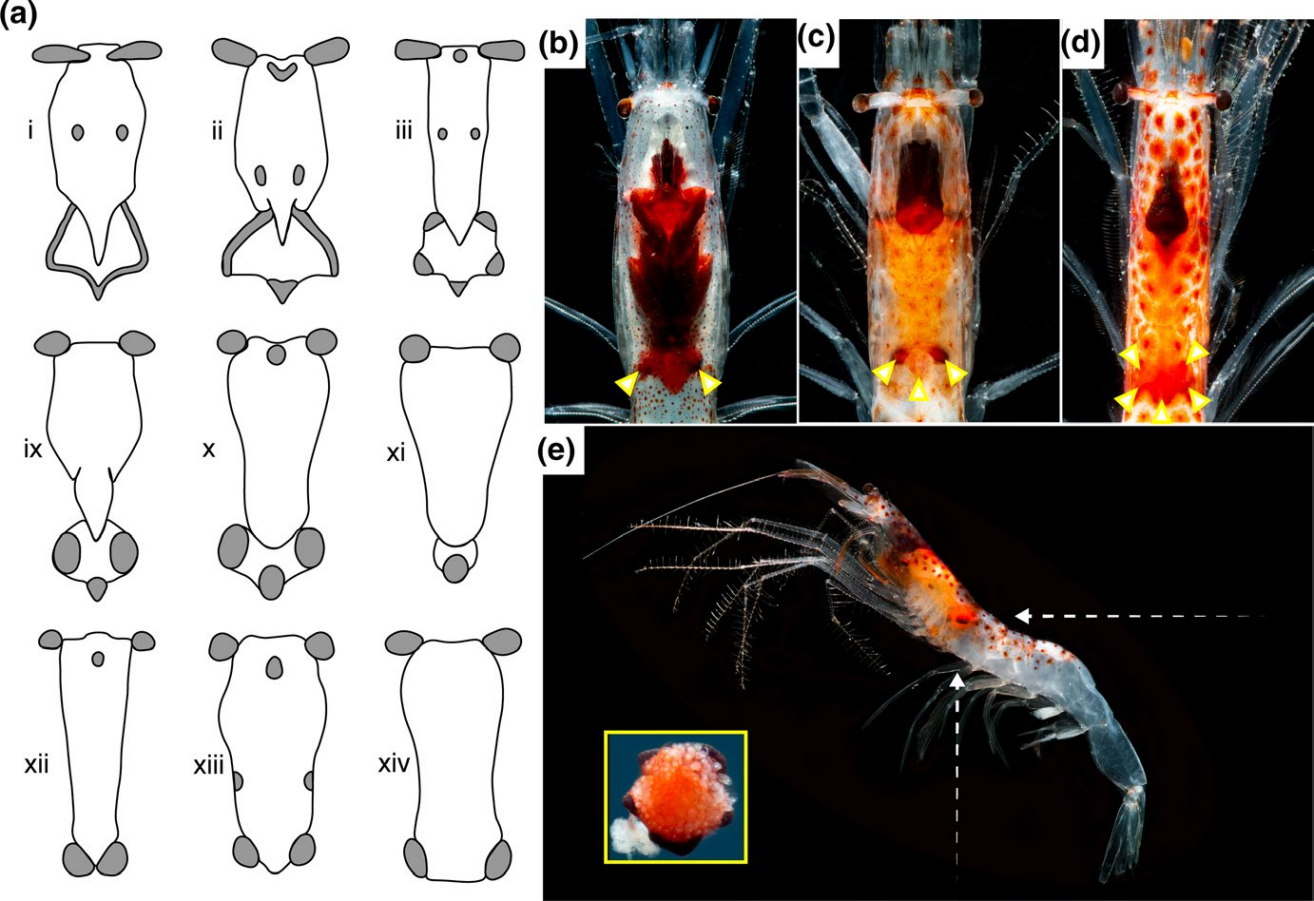
The diversity of light organ (i.e., organ of Pesta) morphologies in family Sergestidae, *Sergestes* sensu lato (s.l.). In this group, organs of Pesta (gray) have diversified across the hepatopancreas (white) for species (a): *Sergestes s.l. arcticus or similis* (i), *corniculum* (ii), *henseni* (iii), *sargassi* (ix), *atlanticus* (x), *cornutus* (xi), *armatus* or *vigilax* (xii), *edwardsii* (xiii), and *pectinatus* (xiv). These figures have been modified from Foxton, 1972. The three species of *Sergestes* s.l. used in this study have distinct posterolateral light organ arrays: *Parasergestes armatus* (b), *Allosergestes sargassi* (c), and *Deosergestes henseni* (d) have bilobed, trilobed, and fringed arrays, respectively (indicated by arrowheads). The organs of Pesta might be viewed in both vertical and horizontal viewing planes in situ (white arrows; e). The species shown in "e" is *Deosergestes henseni* and its dissected posterolateral organs of Pesta are shown (inset). Images used with permission from the photographer, Danté Fenolio (b–e)

Many deep‐sea crustaceans exhibit extensive variation in bioluminescent organ patterns, making them an excellent model for testing the role of bioluminescence in conspecific recognition. The family Sergestidae is a group of deep‐sea shrimps that have species‐specific differences in light organ arrangement, prominent enough to provide characters for taxonomic identification (e.g., Foxton, 1972; Vereshchaka, 1994; Figure 1). Within the Sergestidae exists two subgroups containing multiple genera: the *Sergia* sensu lato (s.l.; Vereshchaka, 2000) and the *Sergestes* s.l. (Vereshchaka, 2009). Unlike the photophores of *Sergia* s.l., the light organs of *Sergestes* s.l. are large, unlensed modifications of the hepatopancreas known as organs of Pesta (Denton, Herring, Widder, Latz, & Case, 1985; Herring, 1981). These organs are predicted to support counterillumination, wherein the production of light replaces the downwelling environmental light blocked by their body, ultimately making them less vulnerable to predation (Johnsen, 2014; McFall‐Ngai, 1990). This function is inferred from behavioral studies of *Sergestes similis* showing that organ of Pesta emissions can match intensity changes in downwelling light (Warner, Latz, & Case, 1979) and that the organs can adjust their angle of tilt to maintain a ventral‐facing direction (Latz & Case, 1982). The predicted function of counterillumination, however, has no obvious requirement for the morphological diversity of light organs in this group, leading Foxton (1972) to question: Have organs of Pesta diversified across *Sergestes* s.l. for the purpose of conspecific recognition?

Studies of *Sergestes* s.l. vision suggest their ability to detect the light that is emitted from organs of Pesta. At least for *Sergestes similis*, the peak wavelength of light emission at 469 nm (Widder, Latz, & Case, 1983) falls within the absorbance spectrum of their monochromatic vision, peaking at 495 (Kent, 1997) or 500 nm (Lindsay, Frank, Kent, Partridge, & Latz, 1999). Identical peak absorbances have been recorded in at least two other species of *Sergestes* s.l. (*Eusergestes arcticus* (Krøyer, 1855) and *Deosergestes corniculum* (Krøyer, 1855)), representing visual sensitives that are also known to overlap with the narrow spectrum of downwelling light in the deep sea (Frank & Widder, 1999; Kent, 1997). This uniformity in *Sergestes* s.l. visual sensitivity is unsurprising given the similar ecological strategies of this group (Felder, Álvarez, Goy, & Lemaitre, 2009).

In addition to being monophyletic (as recovered currently by morphological phylogenies; Vereshchaka, Olesen, & Lunina, 2014), *Sergestes* s.l. genera share overlapping depth distributions (Donaldson, 1975; Felder et al., 2009), with species generally occupying the mesopelagic zone (>200 m and <1,000 m) during the day and migrating into the epipelagic zone (<200 m depth) at night. Thought to encounter one another, sergestid shrimps live in sympatry (Felder et al., 2009) and are known to occur in multi‐species swarms, with some species in the Gulf of Mexico found at abundances of >200 individuals per m^2^ (Flock & Hopkins, 1992). Specifically, some species of *Sergia* s.l. (Omori, 1974) and at least one species of *Sergestes* s.l. (*S. similis*; Omori & Gluck, 1979) have been recorded up to 20 individuals per m^3^ (Vereshchaka, 2009) facilitating reproductive behavior that is thought to occur near surface waters at night. With diverse genitalia and clasping organs on males, the *Sergestes* s.l. are thought to reproduce by copulation (Genthe, 1969; Vereshchaka, 2009), suggesting that the act of finding and identifying conspecifics is not only possible, but required by this group.

To test the hypothesis that the organs of Pesta have morphologically diversified in order to serve conspecific recognition, we examined several aspects of vision across three *Sergestes* s.l. species. These species: *Parasergestes armatus* (Krøyer, 1855),* Allosergestes sargassi* (Ortmann, 1893), and *Deosergestes henseni* (Ortmann, 1893) are sympatric (Felder et al., 2009; Flock & Hopkins, 1992), share overlapping depth distributions at least over the first 1,000 m of the water column (Flock & Hopkins, 1992), and have distinct bilobed, trilobed, and fringed organ of Pesta arrangements, respectively (Figure 1b–d). Using these species, our study examines the visual perception of bioluminescence, testing if it occurs over ecologically relevant distances, permits the visual discrimination of light organ pattern, and differs between the sexes (a potential indicator of sexual recognition function; Herring, 2007). To accomplish this, first we tested for sexual dimorphism in eye‐to‐body size scaling relationships. Second, we modeled the visual ranges (i.e., sighting distances) over which intraspecific bioluminescence may be perceived under natural conditions. Third, we measured external eye morphology to predict maximum possible visual acuity to determine whether these animals can resolve light organ pattern for conspecific recognition.

## 2 MATERIALS AND METHODS

### 2.1 Animals

The three species of deep‐sea shrimps used in this study, *Parasergestes armatus, Allosergestes sargassi*, and *Deosergestes henseni*, represent the three major organs of Pesta morphologies (i.e., bilobed, trilobed, and fringed, respectively; Figure 1b–d). The animals for this study were obtained during two deep‐sea research expeditions, the first occurring in the Straits of Florida on May 4–8, 2019, on the *R/V Weatherbird* and the second occurring in the Northern Gulf of Mexico on June 9–22, 2019, as part of a NOAA Ocean Research Exploration expedition on the *R/V Point Sur*. Animals were captured by 1 or 9‐m^2^ tucker trawl over sampling events that occurred both day and night over a total range of depths from 150–1,500 m. Animals captured on the first expedition were immediately placed in 4% paraformaldehyde in 0.1 M phosphate‐buffered saline (PBS; Boston BioProducts, Ashland, MA, USA) for a 48‐hr fixation step prior to transfer into 0.1 M PBS for subsequent analyses. Those from the second expedition were examined immediately, allowing us to obtain morphological data from fresh animals prior to fixation.

### 2.2 Allometry

Eye diameter and body length were measured for individuals of each species using Mitutoyo CD‐8 ASX Digimatic Calipers (Mitytoyo Corporation, Kanagawa, Japan). Measurements were taken from 19 *Parasergestes armatus* (11 female, 8 male), 29 *Allosergestes sargassi* (19 female, 10 male), and 47 *Deosergestes henseni* (21 female, 26 male). As mentioned, measurements were taken either from fresh or fixed tissue, though the majority were taken from fresh (63% of *P. armatus*, 90% of *A. sargassi*, and 100% of *D. henseni*). For the remaining animals, paraformaldehyde fixation may have resulted in tissue shrinkage, though this may have been applied to both the eye and body proportionally, as plotting eye size as a function of body length (from fresh and fixed tissue together) did not reveal outlying data (Figure S1). Following Hiller‐Adams and Case (1988), eye diameter was measured as the maximum diameter found perpendicular to the dorsoventral eye axis, and body length was measured from the posterior limit of the eye orbit to the end of the telson, which is considered a more reliable measure of body size than carapace length as carapace morphology can differ considerably between species (Hiller‐Adams & Case, 1988). To examine this relationship ourselves, we also measured from the posterior limit of the eye orbit to the end of the carapace (carapace length; Figure S2).

First, we examined eye diameter (*E*) to body length (*BL*) scaling relationships by log transforming the data and fitting it to the power function *E* = *α*(BL)*^β^* (Huxley, 1932). The slope (i.e., allometric scaling factor; *β*) was used to examine the regressions of males and females of each species, with values <1, >1, or = 1 indicating either negative, positive, or isometric scaling, respectively. In line with Diaz, Smith, Serafy, and Ault (2001), differences in eye‐to‐body size scaling between the sexes (i.e., slope (*β*) and intercept (*α*)) were examined by analysis of covariance (ANCOVA; Sokal & Rohlf, 1981) using R (v. 3.4.4; R Core Team, 2018). Critical alpha level was set to 0.016 for multiple testing using the Bonferroni method (Dunn, 1961).

### 2.3 Sighting distance

We used a computational approach originally developed by Nilsson, Warrant, and Johnsen (2014), and then modified by Ruxton and Johnsen (2016), that incorporates information on eye aperture, retinal physiology (i.e., integration rate), and optical properties of the environment and visual targets (here, the organs of Pesta) to estimate the maximum distance at which *Sergestes s.l*. genera can detect intraspecific bioluminescence. In this case, advantages of the Nilsson *et al*. model are (a) that it directly calculates sighting range, (b) that it explicitly takes absorption and scattering by the medium into account, which can of course be critical for aquatic species, and (c) that by assuming optimal summation, it derives the maximal possible sighting distance, which—if short, as is found in this study—suggests that the sighting distance can never be long, even assuming other models.

Though Sergestes s.l. shrimps are thought to spawn in shallow waters at night (Omori & Gluck, 1979), the details of their courtship behavior remain unknown; thus, our analyses represent the maximum possible sighting distances over which shrimps may detect intraspecific bioluminescence. Organ of Pesta patterns are visible from the shrimps’ ventral and dorsal sides, making sighting of bioluminescence patterns possible from at least two perspectives (Figure 1e). In our model, we considered a lighting condition of darkness (i.e., no ambient light) to approximate the conditions of deep water or the epipelagic zone with a new moon phase to yield maximum possible ranges of bioluminescence detection. As reported bioluminescence emission intensities of sergestid shrimps are limited (e.g., Warner et al., 1979), we calculated sighting distances over a wide range of possible emission intensities that have been previously reported for deep‐sea fauna (10^8^, 10^9^, 10^10^, 10^11^ photons/s; Mensinger & Case, 1990; Herring, 2000). Note, the maximum emission intensity necessary to replace light blocked by the opaque viscera within the shrimps’ body for effective counterillumination is also contained within that range.

Our sighting distance model for a bioluminescent point source is shown below (for full derivation see supplemental materials):

$$r=\frac{2}{c}W\left[\frac{\mathrm{cA}}{8\sqrt{3}}\sqrt{\frac{E\Delta t}{1+\sqrt{1+\left(2.8d^{2}N_{0}\right)\Delta t}}}\right]$$

where sighting distance (*r*) is a function of the beam attenuation coefficient of water at 480 nm (*c*), aperture diameter (*A*), photoreceptor diameter (*d*), emittance of the bioluminescent point source (*E*), integration time of the eye (∆*t*), and the photons absorbed per second for a one meter wide aperture in the sighting direction of interest (*N*_0_), which is set to zero because we are considering water with almost no light. The Lambert‐W function (the inverse function of *y* = *xe^x^*) is represented by (*W*). Parameter values for the model were derived from both literature values and original data presented here. The photons absorbed per second from the background light was set to zero. The beam attenuation coefficient was assumed to be constant for depths >200 m and was taken from Ruxton and Johnsen (2016). Photoreceptor diameter was set to 3 µm (Land & Nilsson, 2012). Because aperture diameter is roughly equivalent to eye diameter in *Sergestes* s.l. superposition eyes (Hiller‐Adams & Case, 1988), aperture diameter was set to the mean eye diameter for each species (1.05 mm, 0.72 mm, and 0.83 mm for *D. henseni, A. sargassi,* and *P. armatus*, respectively). We also included a more conservative estimate of aperture diameter that was set to 50% of the mean eye diameter for each species, as the actual aperture size of the shrimp's eyes remain unknown. Integration time for all three shrimps was set to 0.05 s, which was calculated using the average of known critical flicker fusion rate of shrimps in the family Sergestidae (Frank, 2000).

### 2.4 Spatial resolution

We assessed the external morphology of the eye for each species to estimate minimum resolvable angle (*α*_min_), a metric of spatial resolution that indicates the minimum distance that two points remain visually distinguishable. Eyes from two males and two females of each species (*n* = 4 per species) were placed under a Wild Heerbrugg M5 Stereo Microscope (formerly the Wild Heerbrugg Company, Heerbrugg, Switzerland) and imaged using an AmScope MD 35 camera (AmScope, Irvine, CA, USA). Following Baldwin‐Fergus, Johnsen, and Osborn (2015) and Caves, Frank, and Johnsen (2016), images were processed using ImageJ (v 1.38; Schneider, Rasband, & Eliceiri, 2012) to obtain facet diameter (*n* = 10/individual) and the radius of curvature for each eye. Radius of curvature was calculated by fitting circles to the eye image and averaging the circle radii for each individual. Next, we calculated interommatidial angle in radians (Δ*ϕ*), which is the distance between the eye's adjacent ommatidial axes, by dividing the mean facet diameter by the mean radius of curvature. Finally, *α*_min_ was calculated by multiplying Δ*ϕ* by two and converting the unit angle from radians to degrees (Land & Nilsson, 2012). After, we statistically compared the mean minimum resolvable angle between the three species by conducting a one‐way ANOVA as the test assumptions had been met. We used a *post hoc* Tukey HSD test to examine pairwise differences between the groups (*n* = 3).

Based on these calculations of spatial resolution, we estimated how the three species may view each other's organ of Pesta emissions using the *AcuityView* package (v 0.1; Caves & Johnsen, 2018) in R (v. 3.4.4; R Core Team, 2018). Using Fourier methods and acuity of the eye, *AcuityView* removes information from a visual scene that would not be perceptible to animals given their spatial resolution. Here, that visual scene is the shrimps’ organ pattern as seen by a conspecific. Actual images of the shrimps were not used in *AcuityView* as the organs become less visible as the shrimps’ bodies degrade post capture. Instead, we generated images for *AcuityView* by tracing a photograph of each species using Adobe Illustrator, providing realistic outputs of organ proportion and placement. Prior electrophysiological studies have suggested that vision in *Sergestes* s.l. shrimps is monochromatic and spectrally sensitive to the blue‐light emissions of their bioluminescence (Frank & Widder, 1999; Lindsay et al., 1999). Thus, we presented the carapace of the shrimps (made opaque by the viscera) and their luminescent organs of Pesta as black and white, respectively.

In our generated images, the brightness contrast of the light organs likely differs from the brightness contrast that occurs in situ. This difference is irrelevant here as *AcuityView* operates irrespective of brightness contrast, modifying the resolution of scene to reveal what spatial information is available to a given viewer (Caves & Johnsen, 2018). Brightness contrast however, determines the distance over which an object can be detected in situ and thus, organ brightness was factored into our models of bioluminescence sighting distance (see above). For our images, angular size was estimated based on body size data for adults and the viewer was set at an arbitrary distance of 2 cm from the subject. A separation of 2 cm represents a minimum possible distance that the organs of Pesta may be visualized prior to making physical contact and thus, a best‐case scenario over which the shrimps may view each other's light organ patterns. As the actual minimum distance that shrimp may visualize each other remains unknown, we repeated the analysis at distances of 1 and 4 cm. Had we found that the shrimps could achieve pattern discrimination at 1–4 cm, the analysis would have been repeated at greater distances. Finally, the *AcuityView* outputs—reflecting the spatial resolution estimates of each species—were visually assessed and compared.

## 3 RESULTS

### 3.1 Eye‐to‐body size scaling

**FIGURE 2:**
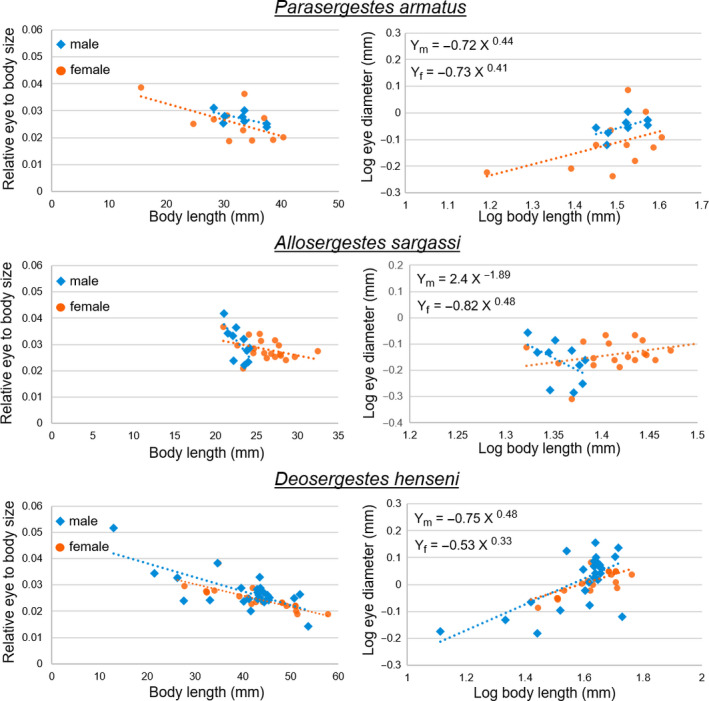
Eye‐to‐body size scaling relationships by sex for three species of sergestid shrimps. Eye size is eye diameter and, like body length, was measured in mm. On the left side, relative eye size is expressed as a function of body length. On the right side, raw eye diameter was plotted as a function of body length on a log–log scale. The allometric equations (Huxley, 1932) for each sex are shown: *Y_m_* and *Y_f_* indicate male and female equations, respectively

A summary of the data including mean body lengths, eye morphometrics, and minimum resolvable angle (*α*_min_) are shown in Table 1. The size ranges obtained for each species were variable, spanning 15–40 mm for *P. armatus*, 21–32 mm for *A. sargassi*, and 13–58 mm for *D. henseni*. On average, the individuals measured for *D. henseni* were largest, having a mean body length of 41 mm (±9 *SD*), relative to 32 mm (±5 *SD*) for *P. armatus* and 25 mm (±3 *SD*) for *A. sargassi*. Eye diameter also followed this trend, with *D. henseni* having the largest mean diameter, 1.1 mm (±0.2 *SD*), while *P. armatus* and *A. sargassi* had mean diameters of 0.8 mm (±0.2 *SD*) and 0.7 mm (±0.1 *SD*), respectively. Plotting the log eye‐to‐body size scaling relationships revealed that eye size increased as body length increased for all groups except *A. sargassi* males, which underwent a reduction in eye diameter during growth (*β* = −1.89; Figure 2). For all groups however, relative eye size scaled negatively with body size (all slope coefficients were <1; Figure 2; Table 2), indicating that relative eye size is reduced as these shrimps grow.

**TABLE 1:** Morphological measures and calculated minimum resolvable angle (mean ± *SD*) for three species of sergestid shrimps

	*Parasergestes armatus*		*Allosergestes sargassi*		*Deosergestes henseni*	
	♂	♀	♂	♀	♂	♀
Eye diameter (mm)	0.9 ± 0.1 (*n* = 8)	0.8 ± 0.2 (*n* = 11)	0.7 ± 0.1 (*n* = 10)	0.7 ± 0.1 (*n* = 19)	1.1 ± 0.2 (*n* = 26)	1.0 ± 0.1 (*n* = 21)
Body length (mm)	32 ± 3 (*n* = 8)	32 ± 7 (*n* = 11)	23 ± 1 (*n* = 10)	26 ± 3 (*n* = 19)	40 ± 9 (*n* = 26)	42 ± 9 (*n* = 21)
Eye‐to‐body size ratio	0.03 ± 0.00 (*n* = 8)	0.03 ± 0.01 (*n* = 11)	0.03 ± 0.01 (*n* = 10)	0.03 ± 0 (*n* = 19)	0.03 ± 0.01 (*n* = 26)	0.03 ± 0.00 (*n* = 21)
Radius of curvature (mm)	0.3 ± 0.02 (*n* = 4)		0.4 ± 0.03 (*n* = 4)		0.5 ± 0.06 (*n* = 4)	
Eye facet diameter (μm)	26.0 ± 4.1 (*n* = 4)		27.2 ± 4.2 (*n* = 4)		23.5 ± 3.2 (*n* = 4)	
Minimum resolvable angle (deg)	10.8 ± 2.1 (*n* = 4)		8.5 ± 1.0 (*n* = 4)		5.9 ± 0.1 (*n* = 4)	

**TABLE 2:** Relationships between eye diameter (*E*) and body length (*BL*) for the sexes of sergestid shrimps

Part 1				
Species	Sex	*n*	*α*	*β*
*Parasergestes armatus*	♂	8	−0.72	0.44
	♀	11	−0.73	0.41
*Allosergestes sargassi*	♂	10	2.4	−1.89
	♀	19	−0.82	0.48
*Deosergestes henseni*	♂	26	−0.75	0.48
	♀	21	−0.53	0.33

Part 2				
Species	Coefficient	*F*	*df*	*p*
*Parasergestes armatus*	*α*	2.569	1	.13
	*β*	0.002	1	.96
*Allosergestes sargassi*	*α*	0.253	1	.62
	*β*	5.221	1	.03
*Deosergestes henseni*	*α*	1.145	1	.29
	*β*	0.77	1	.39

Note

The coefficients *α* (intercept) and *β* (slope) are the parameters of the power function *E* = *α*BL*β*. Part 1 of the table indicates the function coefficients, and Part 2 indicates their statistical relationship by analysis of covariance (ANCOVA). Critical α‐level has been set to 0.016 for a multiple comparison correction using the Bonferroni method (Dunn, 1961)

The log eye‐to‐body size scaling relationships between the sexes of each species were compared by ANCOVA as test assumptions (i.e., sample independence, residual normality, and homoscedasticity) were met. For all three species, no differences were found among the slope or intercept coefficients of males and females.

### 3.2 Bioluminescence sighting distance

Across all model conditions, *D. henseni* had the greatest sighting distance estimates of intraspecific bioluminescence, followed in order by *P. armatus* and *A. sargassi* (Table 3). Across species, average sighting distances modeled for eye aperture equivalent to eye diameter equaled 2.4 m and for the more conservative aperture estimate of half eye diameter, sighting distance averaged 1.27 m. Across considered emission intensities, maximum possible sighting distances for the three species were 6.18, 5.45, and 7.57 m for *P. armatus*, *A. sargassi*, and *D. henseni*, respectively. In each case, sighting distances across the range of emission intensities considered (10^8^, 10^9^, 10^10^, 10^11^ photons/s) increased exponentially, with the shortest sighting distances (due to the lowest emission intensity and smallest aperture size) averaging 0.12 m across species.

**TABLE 3:** Bioluminescence sighting distance estimates for three species of *Sergestes* sensu lato (s.l.) shrimps

Species	Aperture diameter (mm)	Emission intensity (photons/s)	Sighting distance (m)
*Parasergestes armatus*	0.42	10^8^	0.11
		10^9^	0.36
		10^10^	1.11
		10^11^	3.34
	0.83	10^8^	0.22
		10^9^	0.70
		10^10^	2.15
		10^11^	6.18
*Allosergestes sargassi*	0.36	10^8^	0.10
		10^9^	0.31
		10^10^	0.96
		10^11^	2.90
	0.72	10^8^	0.20
		10^9^	0.61
		10^10^	1.88
		10^11^	5.45
*Deosergestes henseni*	0.53	10^8^	0.14
		10^9^	0.45
		10^10^	1.40
		10^11^	4.14
	1.05	10^8^	0.28
		10^9^	0.89
		10^10^	2.68
		10^11^	7.57

Note

Data indicate sighting distances (or visual ranges; m) over which bioluminescence is viewed by conspecifics against a dark background (with no ambient light). Models were generated considering two different aperature diameters for superposition eyes: half diameter and full diameter, as well as a range of bioluminescence emission intensities over four orders of magnitude.

### 3.3 Spatial resolution and perception of conspecifics

**FIGURE 3:**
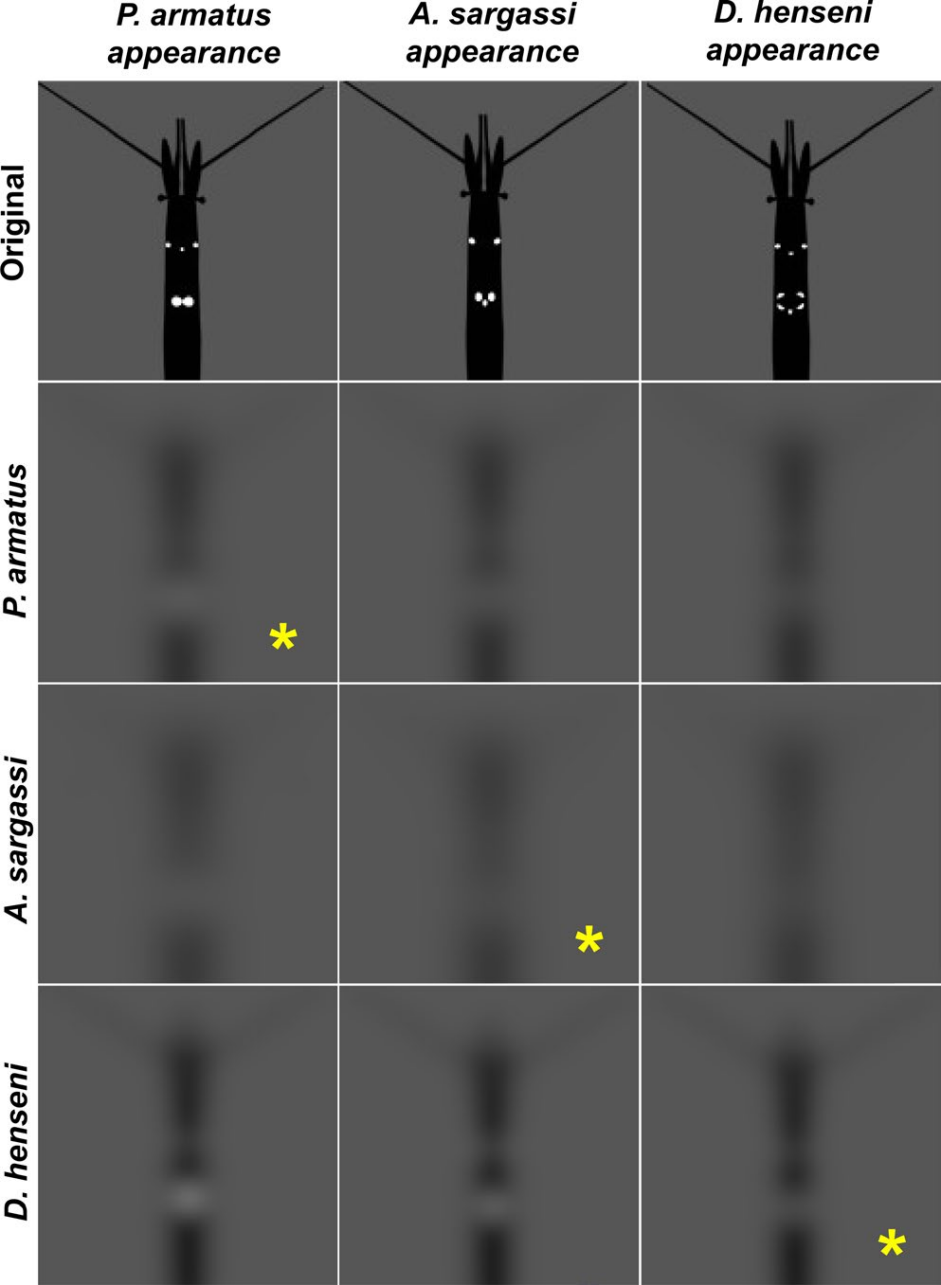
Simulated visual perception of organs of Pesta based on the spatial resolution estimates across the three species: *Parasergestes armatus*, *Allosergestes sargassi*, and *Deosergestes henseni*, which have bilobed, trilobed, and fringed arrays, respectively. Outputs from the *AcuityView* R package (Caves & Johnsen, 2018) are shown, with the appearance of each species presented by column and the spatial information available to their vision (viewed at a distance of 2 cm) presented by row. Yellow asterisks indicate the viewing of conspecifics

Using eye morphology measures, we calculated the mean ± *SD* of minimum resolvable angle (a measure of spatial resolution) for *P. armatus* (10.8 ± 2.1 deg), *A. sargassi* (8.5 ± 1.0 deg), and *D. henseni* (5.9 ± 1.1 deg), which were shown to differ across the groups (*F* = 10.9, *df* = 9, *p* < .01). Specifically, *D. henseni* had a smaller minimum resolvable angle (and thus greater visual acuity) than *P. armatus* alone (*p* < .01), by having the smallest average facet diameter and greatest eye size across all species. Application of these spatial resolution estimates in *AcuityView* allowed us to model how light organ arrangement might be perceived (Figure 3, Figures S3 and S4), which ultimately suggest that *Sergestes* s.l. shrimps cannot resolve organ arrangement of conspecifics, and likely cannot distinguish the organ arrangements found between species. More generally however, the models indicate, that at distances of 1 to 2 cm, the shrimps may have the capacity to detect the collection of light organs (e.g., posterior organs of Pesta) as a singular unit against the carapace (made opaque by the viscera), which may permit the visual detection of other individuals even when bioluminescence intensity is matched to background light during counterillumination.

## 4 DISCUSSION

The results of this study show that *Sergestes* s.l. shrimps appear incapable of resolving variation in organ of Pesta morphology between species, suggesting that bioluminescence may have diversified in this group due to factors other than conspecific recognition. Though *Sergestes* s.l. vision appears unable to discriminate species, detection of bioluminescence emissions over short to moderate distances (~6–7 m at maximum) may help individuals find and approach one another for behaviors that require their proximity, such as aggregation and copulation (Vereshchaka, 2009). Therefore, the extensive diversity of organs of Pesta across this group may serve an alternate function or may result from evolutionary processes such as phenotypic constraint (that is, diversification as a byproduct of design limitations) or neutral drift.

### 4.1 Vision and implications for signaling

To test the hypothesis that light organ variation among *Sergestes* s.l. underlies conspecific recognition, we examined several aspects of *Sergestes* s.l. vision. Across our analyses, the primary morphological feature examined was eye size. Though eyes are metabolically expensive (Laughlin, 2001), increasing eye size can improve both sighting distance and spatial resolution (Cronin, Johnsen, Marshall, & Warrant, 2014); thus, eye size helps indicate the relative importance, and capacities, of a given animal's vision.

Here, we found relative eye size to scale negatively with body length indicating a reduction in relative eye size during growth of *Sergestes* s.l. shrimps. While this occurs commonly in nature and agrees with another study of eye size scaling in deep‐sea shrimps (Hiller‐Adams & Case, 1988), these findings contrast the scaling relationships identified among insects, such as for some hymenopterans, where eye‐to‐body size scaling is isometric (1:1 ratio) (Jander & Jander, 2002) and differs between the sexes (Ribi, Engels, & Engels, 1989). For *A. sargassi* males alone, relative eye size decreased during body growth (slope = −1.89), though this trend did not significantly differ from that of females (slope = 0.48) perhaps due to the low sample size of males (*n* = 10). Future work will determine whether a sexual difference in eye growth exists for this group, but the current data suggest that no sexual dimorphism exists in eye size scaling, providing no support for the importance of vision in *Sergestes* s.l. sexual interaction.

Sighting distance models examining the visual ranges in which these shrimps can detect one another's bioluminescence indicated at maximum that emissions can be detected at <8 m for *D. henseni* and <7 m for the other two species, with an average sighting distance (across species and predicted emission intensities) at 1.83 m. These findings indicate that *Sergestes* s.l. shrimps might find one another over distances greater than several meters using other sensory modalities, such as chemoreception, which is shown to be an important means of communication during sexual and social interactions among decapod shrimps (Bauer, 2011). In crustaceans, chemical signals, such as volatile pheromones and metabolites in urine, are released from the body and detected by other individuals using olfactory receptors located on antennae (Bauer, 2011). Such signals may help *Sergestes* s.l. detect and approach one another before bioluminescent emissions can be seen. These findings are in line with the conclusions of Herring (2000), showing that estimated nearest neighbor distances of many deep‐sea animals (including shrimps) exceeds the distances over which bioluminescent signals can be transmitted, and thus, other sensory systems may be required for initial contact to be made.

The spatial resolution of these shrimps (modeled using *AcuityView* software) indicated that even at very close range (2 cm), *Sergestes* s.l. shrimps appear incapable of discriminating light organ arrangement. *AcuityView* portrays the spatial information of a scene available to a given viewer, but does not account for edge enhancement and other neural processes that may improve the sharpness of the scene (Caves & Johnsen, 2018). While our method cannot account for postprocessing of visual information by the shrimps, their potential to distinguish organ pattern better than what is indicated by *AcuityView* is unlikely due to (a) that morphological approaches, such as those conducted here, can yield inflated estimates of visual acuity relative to those estimated from behavior (e.g., Caves et al., 2016) and (b) the reflecting superposition eye type found in this group, pools spatial information onto individual photoreceptors, yielding actual visual acuities that may be lower than what is reported here. Together with our data, these factors suggest that the spatial resolution of *Sergestes* s.l. shrimp vision is likely insufficient for light organ discrimination between these species.

Our study provides new insights into the visual perception of bioluminescence among mesopelagic shrimps, but has several limitations related to the difficultly of studying life in deep sea. Firstly, though a function of counterillumination is predicted, *Sergestes* s.l bioluminescence has not been studied in situ, making alternative functions (such as antipredation and warning signaling) possible that may not require organ pattern to be resolved. Further, while *Sergestes* s.l. appear incapable of distinguishing species by light organ pattern, temporal differences in light emissions, such as pulses or flashes, may underlie conspecific recognition or communication. However, light pulses or flashes have yet to be reported for this group. Finally, the present analysis operates on several assumptions as information is lacking on the actual emission intensities of *Sergestes* s.l. bioluminescence and the visual ranges over which they observe each other in situ. While future work in these areas may ultimately reveal some visual signaling function of *Sergestes* s.l. bioluminescence, at least from the data presented here, light organ variation is not explained as a signal for conspecific recognition.

## 5 CONCLUSIONS

The findings of this study impart caution about interpreting the significance of visual characters based on the capacities of human vision (Caves, Nowicki, & Johnsen, 2019). Our keen ability to discriminate *Sergestes* s.l. light organ arrangement has led to predictions about its role in conspecific recognition in the past (Foxton, 1972); however as shown here, such hypotheses can only be tested by considering the visual capacities of the viewer.

The question remains, why have *Sergestes* s.l. undergone species‐specific diversification in organ of Pesta arrangement, if not for conspecific recognition? Other hypotheses include that variations in organ of Pesta may permit effective counterillumination of their differently shaped opaque viscera (Foxton, 1972) or variable pigmentation given by their distributions of chromatophores. Alternatively, organ variation may not serve a biological function, but rather, occur as the byproduct of evolutionary descent. Organ of Pesta diversification may have occurred due to neutral drift, and current work is underway testing this hypothesis by tracing the evolutionary history of light organs across the sergestid tree of life. Another possibility is that organ diversification occurred due to evolutionary constraint, a concept suggesting that physical design limitations or developmental requirements secondarily result in phenotypic diversification of a trait (Arnold, 1992). Many deep‐sea groups exhibit species‐level differences in bioluminescence, and the *Sergestes* s.l. are just one group showing that a visual signaling function cannot be assumed. More work on this and other groups will help reveal the evolutionary and ecological basis of the extensive bioluminescence variation found among these animals and others in the deep sea.

## CONFLICT OF INTERESTS

The authors have no conflicts of interest to declare.

## AUTHOR CONTRIBUTION

**Lorian E. Schweikert:** Conceptualization (equal); Data curation (equal); Formal analysis (equal); Investigation (equal); Methodology (equal); Project administration (equal); Supervision (equal); Validation (equal); Visualization (equal); Writing‐original draft (equal); Writing‐review & editing (equal). **Alexander L. Davis:** Data curation (equal); Methodology (equal); Visualization (equal). **Sönke Johnsen:** Data curation (equal); Formal analysis (equal); Methodology (equal). **Heather D. Bracken‐Grissom:** Conceptualization (equal); Data curation (equal); Investigation (equal); Project administration (equal); Supervision (equal).

## Supporting information

Appendix S1

## Data Availability

The code for the sighting distance modeling has been publicly archived here: https://github.com/a‐l‐davis/Schweikert‐et‐al.‐2020.git. All other data included in this study is available in the main text and supplemental materials.
