# Supplementary material for: Visual perception of light organ patterns in deep‐sea shrimps and implications for conspecific recognition
Source: Ecol Evol. 2020 Aug 7;10(17):9503–13. doi: 10.1002/ece3.6643 (PMC7487218; doi:10.1002/ece3.6643)
Supplement: Supplementary file 1 — Appendix S1 [file ECE3-10-9503-s001.docx]

**­Supplemental Material**


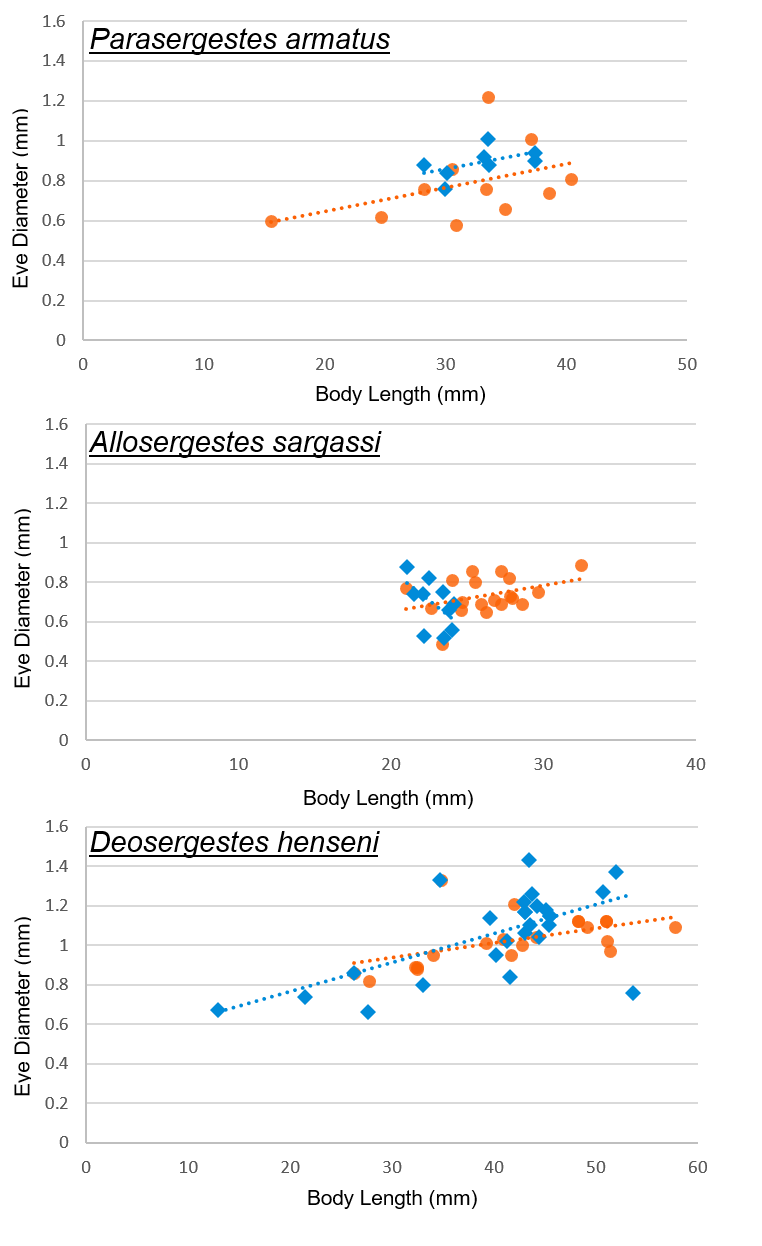


Fig S1. Raw eye diameter to body length scaling relationships by sex for three species of *Sergestes* sensu lato (s.l.) shrimps.


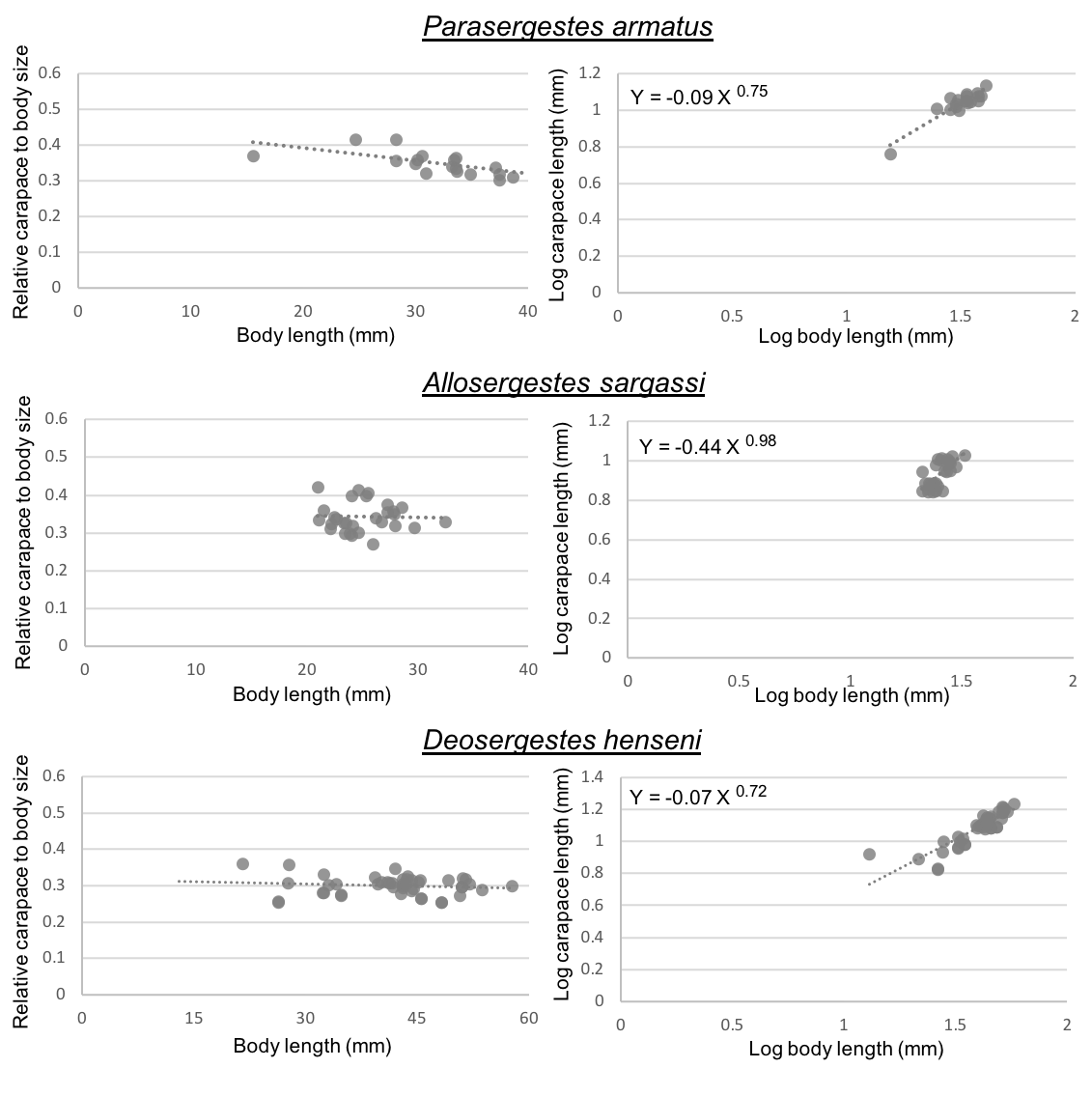


Fig S2. Carapace-to-body size scaling relationships by sex for three species of *Sergestes* sensu lato (s.l.) shrimps. Both carapace length and body length were measured in mm. On the left side, relative carapace size is expressed as a function of body length. On the right side, raw eye diameter was plotted as a function of body length on a log-log scale. The allometric scaling equation (Huxley 1932) for each species is shown. For all species, carapace length scaled negatively with body growth (β < 1), lending further support that body length provides a more appropriate measure (than carapace length) for allometric comparisons.


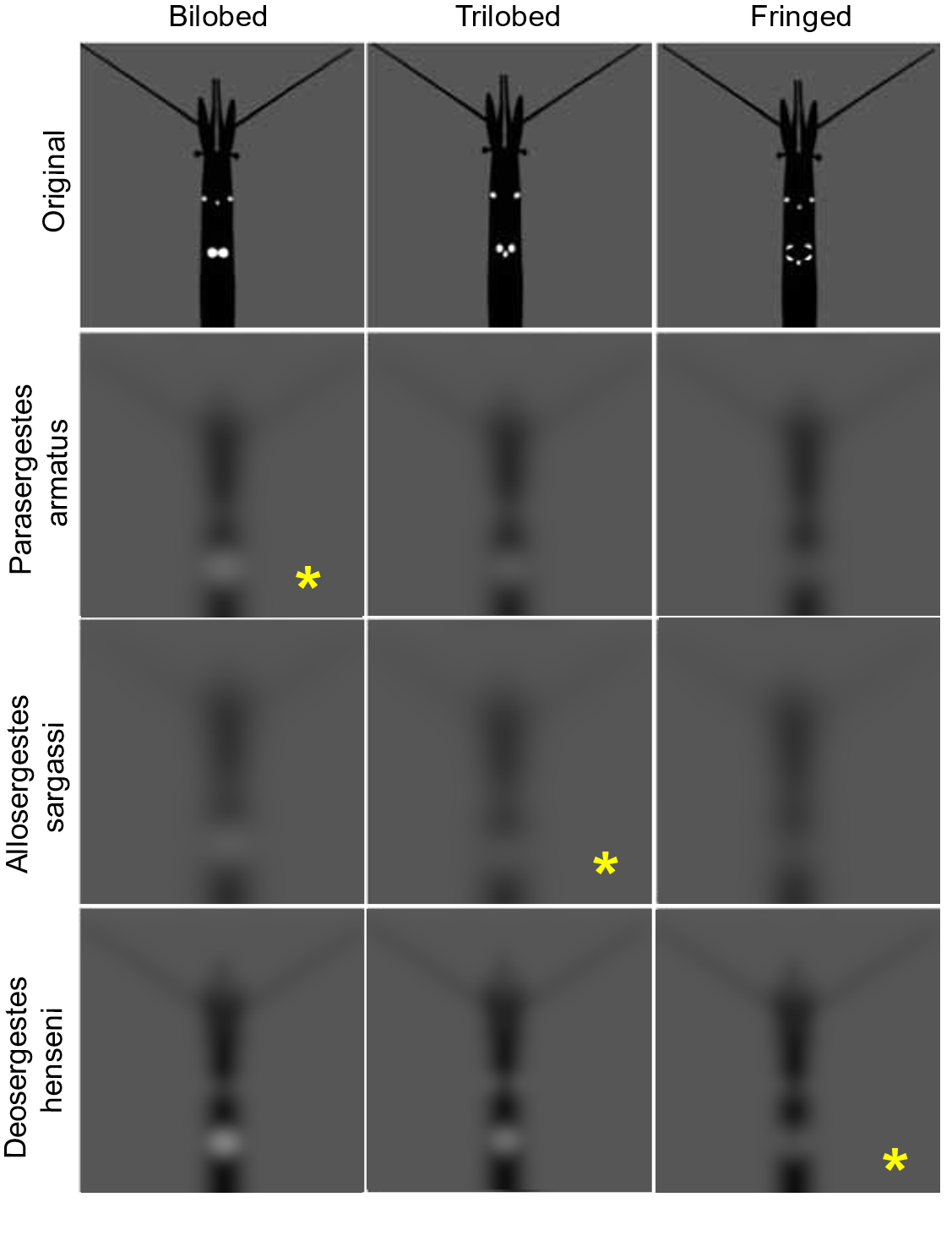


Fig. S3. Simulated visual perception of *Sergestes* sensu lato (s.l.) organs of Pesta based on the spatial resolution estimates across the three species: *Parasergestes armatus*, *Allosergestes sargassi*, and *Deosergestes henseni*, which have bilobed, trilobed, and fringed arrays, respectively. Outputs from the *AcuityView* R package (Caves & Johnsen 2018) are shown, with the appearance of each species presented by column and the spatial information available to their vision (viewed at a distance of 1 cm) presented by row. Yellow asterisks indicate the viewing of conspecifics.


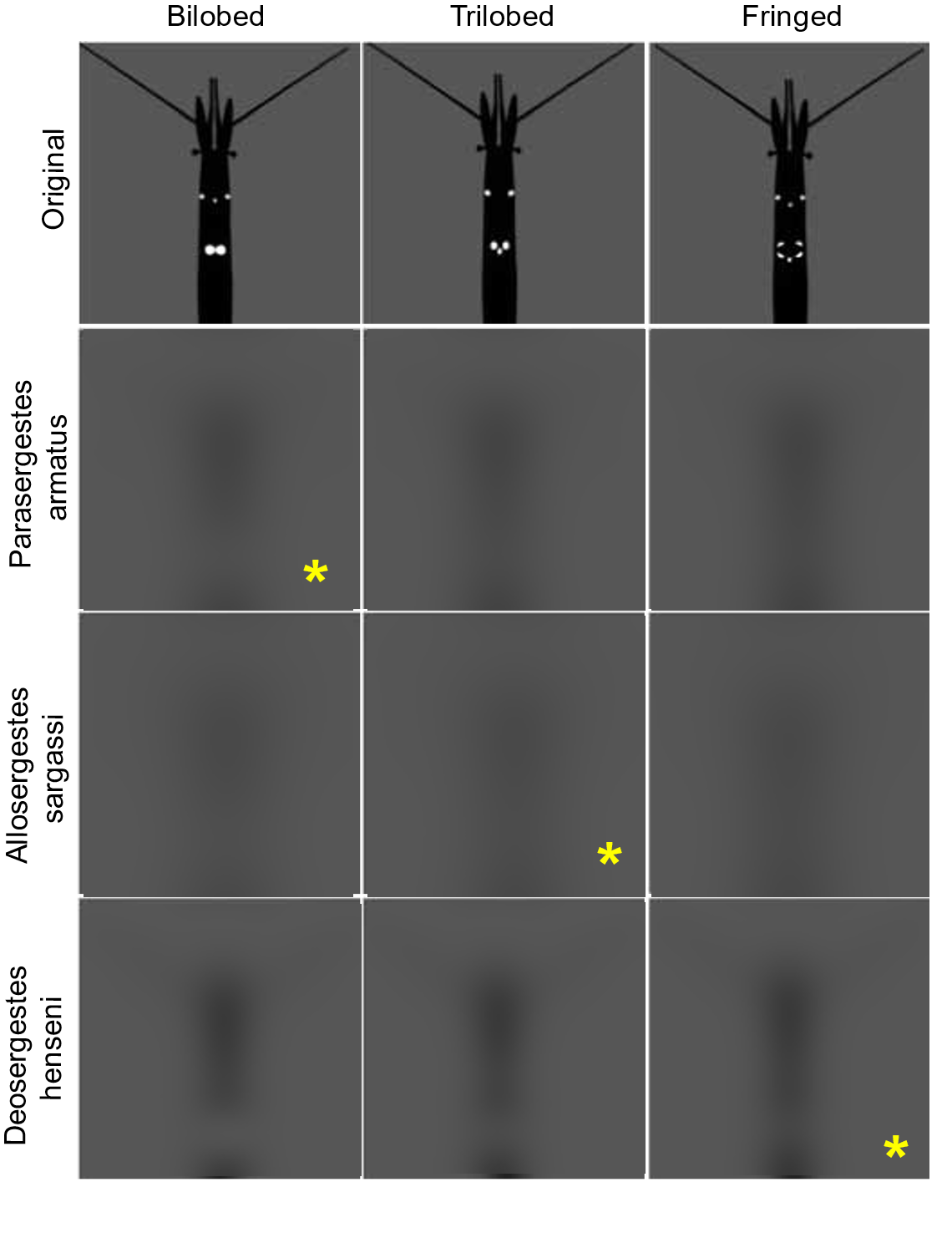


Fig. S4. Simulated visual perception of *Sergestes* sensu lato (s.l.) organs of Pesta based on the spatial resolution estimates across the three species: *Parasergestes armatus*, *Allosergestes sargassi*, and *Deosergestes henseni*, which have bilobed, trilobed, and fringed arrays, respectively. Outputs from the *AcuityView* R package (Caves & Johnsen 2018) are shown, with the appearance of each species presented by column and the spatial information available to their vision (viewed at a distance of 4 cm) presented by row. Yellow asterisks indicate the viewing of conspecifics.

Derivation for the Sighting Distance Models

We begin with the following definitions:

number of photons collected from one point source in one integration time


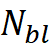

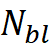


number of photons collected from the background water in one integration time (over a pixel the same angular diameter as the pixel viewing the point source)


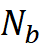

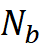


*X_ch_* dark noise

The pelagic light field is approximately monochromatic at viewing angles greater than 48° from vertical (i.e. outside Snell’s window) even at relatively shallow depths and at all viewing angles at depths greater than ~100 m. In these situations, the beam attenuation coefficient of the water (*c*) can be considered to be approximately constant and equal to the value at the wavelength of peak penetration (480 nm in this study).

The number of photons from the point source is . Suppose you want to have a 50% chance of detecting the source, with a 2.5% chance of a false alarm. Then the sensitivity index must equal , where Z is the inverse of the cumulative normal distribution function (Macmillan and Creelman 2004). Because the variance of photon counts (and dark noise) equals the mean, the sensitivity index also equals:


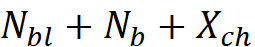

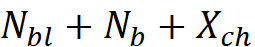

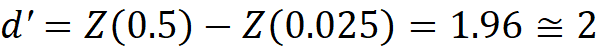

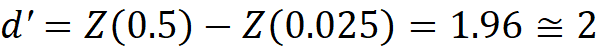


. (1)


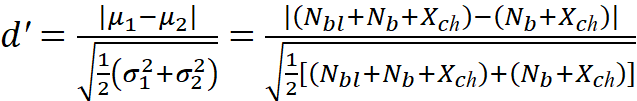

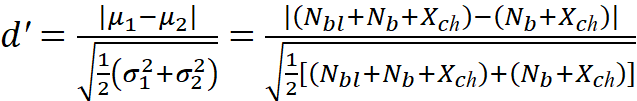


Therefore:

(2)


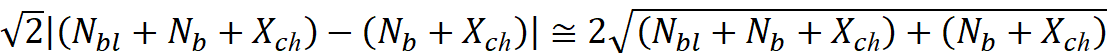

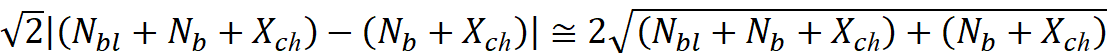


Equation (2) assumes that the BL source is so small that much of the viewer’s pixel is filled with background light. For this reason, equation (2) also does not include pathlight. This reduces to:

(3)


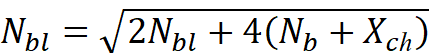

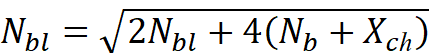


Squaring both sides and rearranging gives:

, (4)


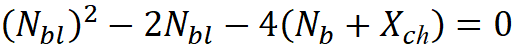

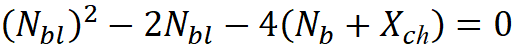


which can be solved for *N_bl_* as:

(5)


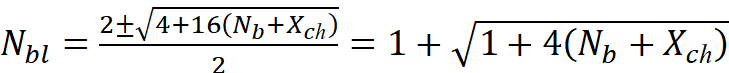

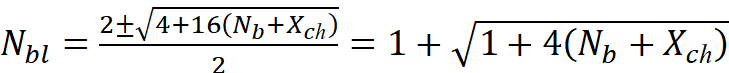


From Nilsson et al., 2014:

, (6a)


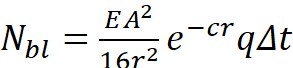

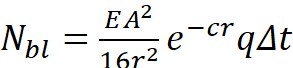


, and (6b)


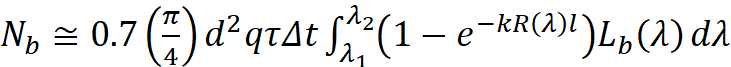

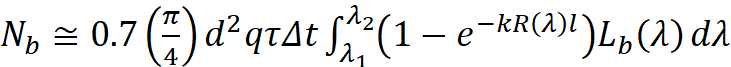


(6c)


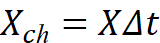

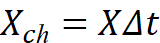


For the viewing organism *A*, *q*, *d*, *τ*, and Δ*t* are the diameter of the pupil, the quantum efficiency of the photoreceptors, the width of the photoreceptors, the ocular transmittance, and the integration time of the photoreceptors respectively. The parameters *k* and *l* are the absorption coefficient and the length of the photoreceptors respectively. *L_b_* (*λ*) is the spectral radiance of the background light and *R*(*λ*) is the normalized absorbance spectrum of the photoreceptors. *E* is the emittance of the bioluminescent source (in all directions in photons/s) and *c* is the beam attenuation coefficient of the water. *X* is dark noise per photoreceptor. For convenience, we define:

(7)


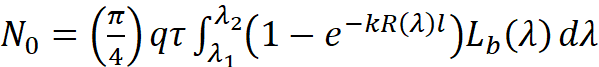

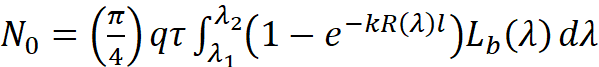


which is the number of photons absorbed in one second by a pixel that views a region one steradian in angular area (divided by 0.7, so that we can use the tables we already have for extended viewing scenarios). This can be thought of as the product of the sensitivity of the eye and the amount of light available for vision. Since the terms can not be separated, due to the weighted integral, they are considered as one. Thus, . Substituting equations (6a-6c) into equation (5), and assuming that *q* is 0.33, gives:


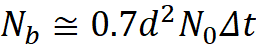

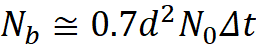


(8)


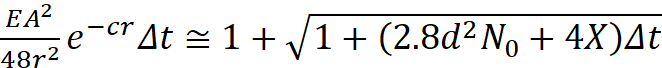

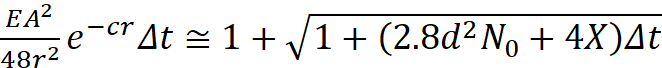


Rearranging gives:

(9)


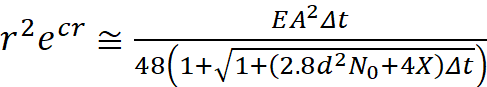

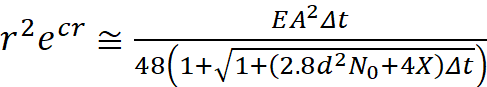


The square root of this is:

(10)


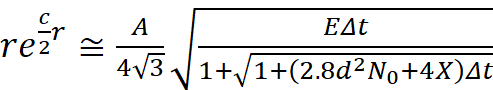

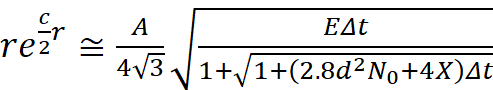


This can be solved for *r* as:

, (11)


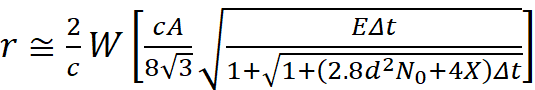

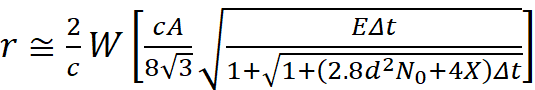


where W is the lambertW function (inverse of y = xe^x^).

Because *X* is small relative to the number of photons, this can be simplified further to:

(12)


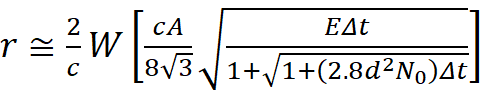

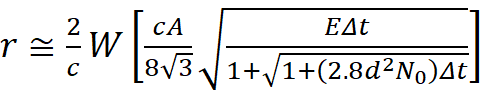


References:

Macmillan, N.A. and Creelman, C.D., 2004. *Detection theory: A user's guide*. Psychology press.

Nilsson, D.E., Warrant, E. and Johnsen, S., 2014. Computational visual ecology in the pelagic realm. *Philosophical Transactions of the Royal Society B: Biological Sciences*, *369*(1636), p.20130038.
